# Challenges and Successes in Introducing Coronary CT Angiography in an Emergency Medicine-Run Observation Unit

**DOI:** 10.7759/cureus.63620

**Published:** 2024-07-01

**Authors:** Catherine Williams, Matthew J Van Ligten, Brianna Tomlinson, Robert Kelly, Charis E Wang, Erin Root, Matthew J Swan, Nicole R Hodgson, Douglas Rappaport, Wayne A Martini

**Affiliations:** 1 Department of Emergency Medicine, Mayo Clinic Alix School of Medicine, Scottsdale, USA; 2 Department of Hospital Internal Medicine, Mayo Clinic Arizona, Phoenix, USA; 3 Department of Emergency Medicine, Mayo Clinic in Arizona, Phoenix, USA

**Keywords:** acs (acute coronary syndrome), chest pain, observation medicine, angina, coronary artery disease, coronary ct angiography, ct coronary aniography, noninvasive cardiac evaluation, provider education and collaboration, nursing education

## Abstract

This study was designed to analyze the departmental changes in transitioning the Emergency Department (ED)-run Observation Medicine Unit's routine noninvasive cardiac evaluation from the traditional standard-of-care procedures to coronary computed tomography angiography (CCTA).

While the routine use of CCTA for the evaluation of chest pain has been deemed feasible and safe, provider confidence appears apprehensive, and ordering patterns appear reluctant to change.

We conducted a retrospective analysis of data from two risk-matched cohorts of ED patients who presented with symptoms suggestive of acute coronary syndrome (ACS) but without ischemic electrocardiogram (ECG) changes or positive troponin. Endpoints included length of stay, major adverse cardiovascular event (MACE) rates at 28 days, recidivism rate, and downstream findings on coronary catheterization.

The adoption of CCTA led to a significant reduction in the length of stay for patients in the ED-run Observation Medicine Unit. Provider and nursing education initiatives were crucial in overcoming initial resistance and improving the implementation of CCTA. Post-education, there was a marked increase in the volume of CCTA performed and a decrease in the length of stay, enhancing overall departmental throughput.

The results suggest that CCTA offers a reliable and efficient diagnostic alternative to traditional noninvasive tests, with high diagnostic accuracy contributing to faster decision-making and reduced need for invasive procedures. Continuous education for providers and nursing staff was essential to ensure adherence to the new protocol and improve clinical outcomes.

Transitioning to CCTA for routine noninvasive cardiac evaluation in the ED-run Observation Medicine Unit demonstrated significant efficiency and diagnostic accuracy benefits. Successful implementation requires targeted educational efforts to ensure competency and confidence among healthcare providers. The findings support the integration of CCTA into standard clinical practice for the evaluation of chest pain in the emergency setting, with future research needed to validate these results in broader patient populations and assess long-term outcomes.

## Introduction

Evaluation and management of patients presenting to the Emergency Department (ED) with chest pain represents a critical challenge to Emergency Medicine. Accurate and efficient diagnostic strategies are critical for identifying patients at risk and ensuring timely intervention. In addition to ruling out myocardial infarction in the ED by doing serial electrocardiogram (ECG) and cardiac biomarker measurements, noninvasive tests, which include exercise treadmill ECG testing (ETT), stress echocardiography (echo), and rest or stress myocardial perfusion imaging with Tc-99m (SPECT) are often done to help identify at-risk patients and identify patients who would potentially benefit from urgent coronary catheterization.

These noninvasive tests exhibit commendable sensitivity levels (ETT 76%, SPECT 83%, and echo 85%) [1-3] in detecting significant coronary artery stenosis, especially when compared to the gold standard of coronary angiography. However, their specificity remains moderately effective (ETT 60%, SPECT 64%, and echo 77%). Consequently, due to these relatively modest specificities, 33% to 44% of patients with suspected acute coronary syndrome (ACS) who undergo cardiac catheterization ultimately do not exhibit significant coronary artery disease (CAD) [4,5].

Traditionally, the standard evaluation process for patients with chest pain, which incorporates stress testing, typically necessitates hospital admission lasting between 24 and 36 hours, a practice observed in over 90% of healthcare facilities in the United States [6].

There is a strong consensus within the medical community that coronary computed tomography angiography (CCTA) represents a powerful diagnostic tool. CCTA’s ability to reliably exclude the presence of coronary stenosis and clinically significant coronary plaque is demonstrated by its impressive negative predictive value (NPV) ranging from 95% to 98% and positive predictive value (PPV) ranging from 92% to 97% [7]. This performance exceeds that of standard noninvasive tests, while also reducing the length of stay by an average of 7.6 hours [8]. A systematic review has also found that using CCTA was a safe and cost-effective method of screening patients with low-to-intermediate-risk chest pain, due to similar rates of adverse events, a reduction in length of stay by 17%, and a reduction in short-term costs of 21% [9].

While proven to be an effective and cost-efficient screening tool, the use of CCTA in patients with angina represents challenges to those trying to implement the use of the screening tool. For example, it is known that the quality of the CCTA is affected by fast heart rate, obesity, and/or arrhythmia [10]. Given these drawbacks, the use of CCTA as a screening tool may require treatments such as the administration of a beta-blocker for rate control in an otherwise healthy, non-tachycardic patient. While generally safe if used cautiously, the administration of medications like these for this test may pose a challenge to nursing and advanced practice providers who are considering adopting this new and effective screening tool.

The goal of this study is to review the challenges to practice changes within our department, education for nursing and advanced practice providers running the Observation Medicine unit with supervision, and other practice changes that have impacted the improvement of departmental throughput. 

## Materials and methods

Study design

From March 1, 2023, to March 1, 2024, the Emergency Medicine Observation Unit treated 271 patients with symptoms suggestive of ACS. The inclusion criteria for ordering CCTA were based on consensus among the Cardiology, Radiology, and Emergency Medicine Departments.

Participant selection 

**Figure 1:**
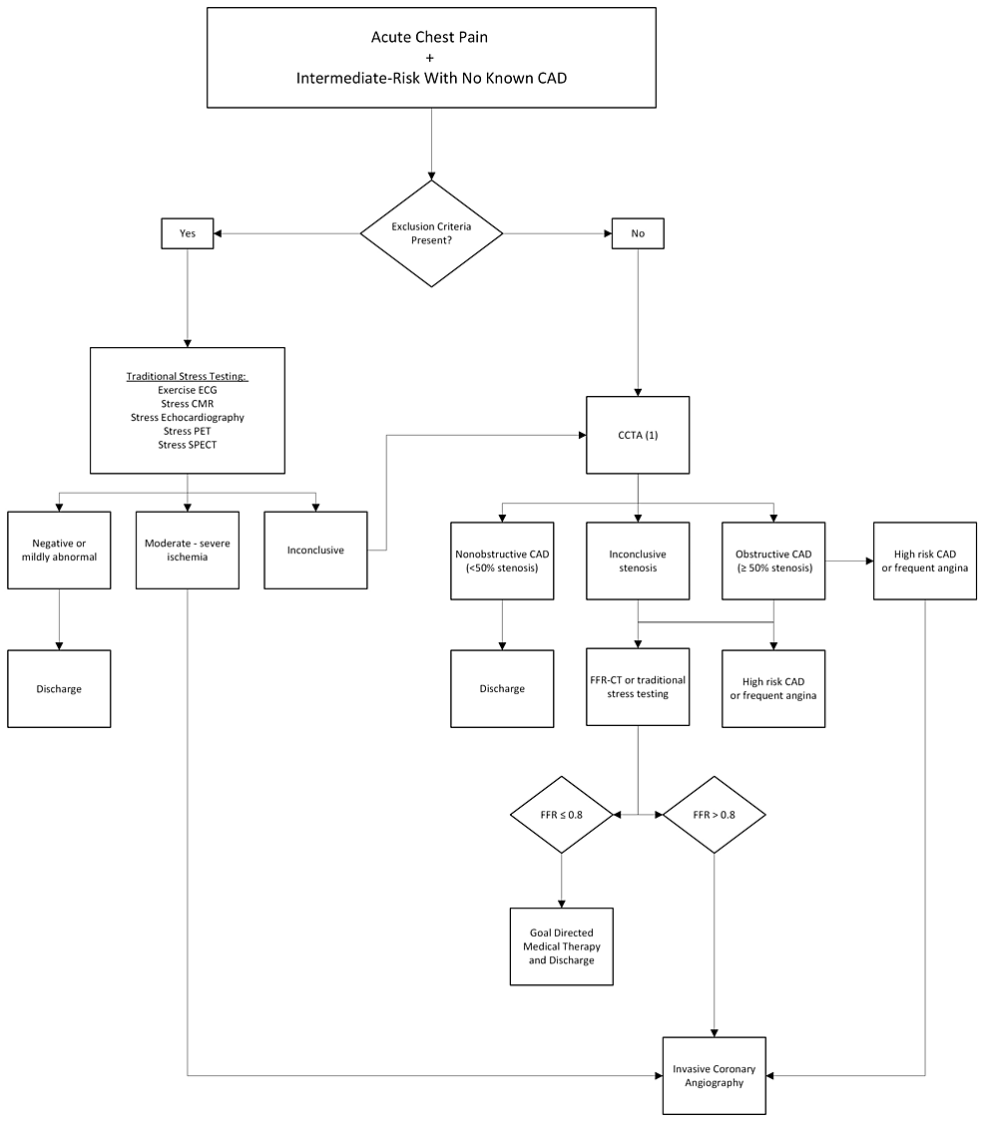
Coronary CT angiography (CCTA) flowchart for inclusion criteria and disposition management to discharge with or without goal-directed medical therapy vs. invasive coronary angiography. CAD, coronary artery disease; FFR-CT, fractional flow reserve-computed tomography; exercise ECG, exercise electrocardiogram; stress CMR, stress cardiovascular magnetic resonance imaging; PET, positron emission tomography; SPECT, single photon emission computed tomography

Patients were considered for CCTA if they met the criteria for intermediate risk based on the HEART Score and met certain diagnostic criteria (Figure 1). All scans were performed using a multi-slice (multi-detector) CT scanner with 256 slices. They were excluded if they were greater than or equal to 65 years of age, had a history of documented CAD, had an abnormal heart rhythm, inability to hold their breath, or had utilized sildenafil (Viagra) or tadalafil (Cialis) due to its medication interaction with nitroglycerin. Patients who did not meet the criteria for CCTA received either an exercise electrocardiogram, stress cardiovascular magnetic resonance imaging, stress echocardiogram, stress positron emission tomography (PET), or single photon emission computed tomography (SPECT) imaging.

Results of CCTA were ranked as nonobstructive CAD (<50% stenosis), inconclusive stenosis, and obstructive CAD. Patients with nonobstructive CAD were discharged. Patients with inconclusive stenosis were sent for fractional flow reserve-computed tomography (FFR-CT).

Patients who had inconclusive findings on CCTA, such as inconclusive stenosis or obstructive CAD without high-risk CAD symptoms or frequent angina, were referred for FFR-CT. FFR-CT combines the anatomical information from CTA with computational fluid dynamics to simulate the blood flow and pressure within the coronary arteries. Patients with a CCTA showing CAD > 50% stenosis who exhibited high-risk CAD symptoms or frequent angina were sent directly for invasive coronary angiography. Patients with an FFR-CT greater than or equal to 0.8 were brought for invasive coronary angiography, while patients with coronary disease less than that were given goal-directed medical treatment for CAD.

Interventions 

The initial adoption of CCTA was slow and required multiple educational opportunities for key players involved in patient care within the ED-run Observation Medicine Unit. The challenges included advanced practice providers' comfort with understanding the clinical utility of the tests, nursing comfort with β-blockade to achieve a heart rate between 50 and 70 beats per minute (BPM) for clinically meaningful imaging, and patient education initiatives to improve comfort with changes from what is traditionally viewed as a stress test.

Initial provider education (January 2023) included reviewing the safety of a negative coronary CT angiogram for identifying patients suitable for discharge from the ED, with low rates of major adverse cardiovascular events, significantly reduced costs, and greater efficiency in terms of time to discharge [11]. Using these principles, we aimed to significantly improve throughput in the observation unit by reducing the volume of patients exceeding the 23-hour threshold goal. A review of initial provider education occurred in June 2023, promoting the utilization of CCTA.

Nursing comfort with beta-blockade provides a secondary challenge. CCTA requires a slow, regular heart rate for optimal image quality. If patients had a resting heart rate ≥ 60 BPM, patients were to receive medication for heart rate control (unless contraindicated). Oral metoprolol was given one hour before the exam. Repeat doses of 50 mg tablets were to be given every 30 minutes if heart rate exceeded 70 BPM and systolic blood pressure was greater than 90 mmHg for a total max dose of 150 mg. Through educational efforts from providers and unit nursing leadership, noncompliance with protocol stagnated. Nursing comfort significantly improved by giving beta-blockers to patients with normal heart rates. 

Finally, intermittent provider re-education on the clinical usefulness of considering patients for CCTA and how it can help with throughput was necessary to help prevent providers from defaulting to traditional stress tests and not filtering patients through the criteria required for CCTA.

## Results

**Figure 2:**
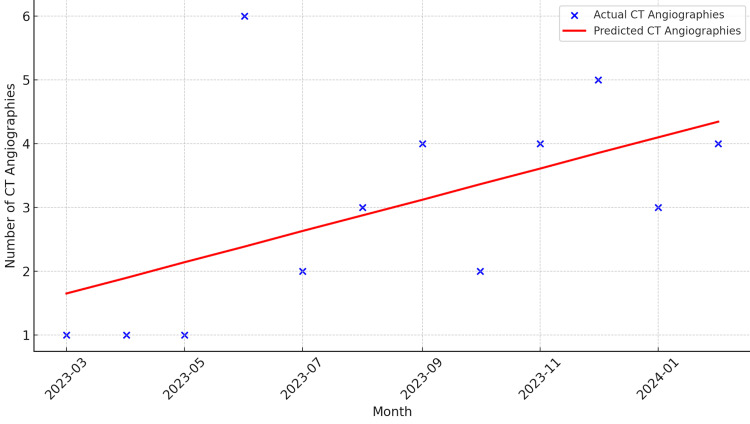
Linear regression of coronary CT angiographies (CCTAs) over time showing increase of 0.2448 CCTA throughout the time period.

**Figure 3:**
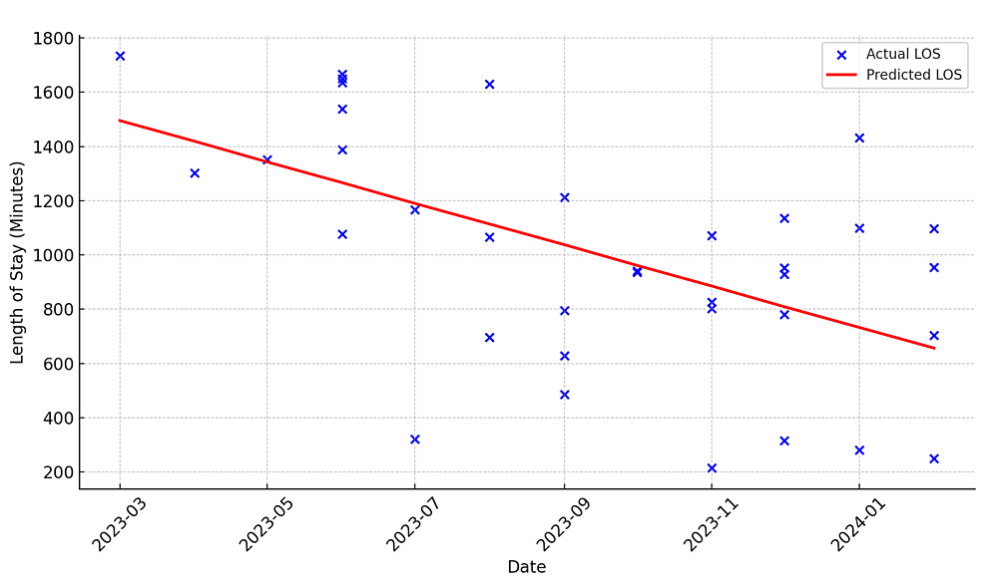
Linear regression model for predicting the length of stay (LOS) showing a slope coefficient of -76.23, indicating an average reduction in LOS of 76.23 minutes per month during this introductory period.

From March 2023 to May 2023, the number of CCTAs in the Observation Medicine Unit remained low, while the average time spent in the observation unit increased compared to subsequent months, suggesting a correlation between underutilization of CCTAs and longer lengths of stay. After providing re-education, there was a sixfold increase in the volume of CCTAs from May to June, and the subsequent length of stay also decreased. We did experience a decrease in the number of CCTAs, which was eventually discovered to be secondary to nursing staff being uncomfortable with the use of beta-blockers in patients with normal or slow heart rates. Due to these concerns, the nursing staff was educated from August to September 2023, and the number of patients receiving CCTAs statistically increased (Figure 2). Regarding the patient’s length of stay, there was a statistical and consistent decrease in the length of stay attributed to the education about the use of CCTAs for nursing and advanced care providers in our observation unit (Figure 3).

CCTA volume and length of stay

The average length of stay in the ED-run Observation Medicine Unit decreased significantly following the increased use of CCTA. Prior to the reeducation efforts, the average length of stay was approximately 20.5 hours. Post-reeducation, the average length of stay reduced to 14.2 hours. This reduction correlates with the increased comfort and proficiency of the providers and nursing staff in using CCTA as a primary diagnostic tool for chest pain evaluation.

Clinical outcomes

The study evaluated the clinical impact of CCTA introduction into the ED-run Observation Medicine Unit. This includes secondary measures such as the rate of MACEs at 28 days, rate of recidivism, and downstream findings for patients who underwent a coronary catherization. All patients had a CCTA performed as their stress test equivalent while in the unit did not have any major adverse cardiac events within the 28-day period. 

Recidivism and follow-up

Four patients returned to the ED within 28 days of their observation stay for chest pain after undergoing CCTA: Patient 1 returned for near syncope with a normal EKG and no further cardiac abnormalities on lab work. Patient 2 returned for chest pain and dizziness, was found to be in atrial fibrillation without signs of ischemia, and was ultimately set up for outpatient ablation. Patient 3 returned for a wound check related to femoral catheter insertion. Patient 4 initially deferred intervention despite moderate stenosis on coronary CT angiography and underwent coronary catheterization on return visit.

Invasive procedures

Five patients were found to have moderate-to-severe risk and underwent catheterization on the same day or the following day, resulting in coronary artery stent placement. One patient was found to have significant aortic stenosis and underwent transcutaneous aortic valve replacement.

Statistical analysis

A statistical analysis was conducted to evaluate correlation between the implementation of CCTA and the patient's length of stay in the Observation Medicine Unit. A t-test revealed a significant decrease in the length of stay (*P *< 0.05) in the last three months compared to the first three months of the introductory period. Additionally, a t-test analyzing the number of CCTAs performed in the first three months and the last three months indicated a significant increase (*P *< 0.01). Two linear regression models were used to analyze the trends over time: The first model revealed the number of CT angiographies, showing a significant upward trend (increase of 0.2448 scans per month), while the second model analyzed the length of stay, indicating a significant downward trend (decrease of 76.23 minutes per month).

In summary, the results of this study demonstrate that the implementation of CCTA significantly increased the volume of CCTAs performed and led to a reduction in the length of stay. These findings suggest that continuous education and training for providers and nursing staff are crucial for the successful implementation of new diagnostic protocols such as CCTA, resulting in improved efficiency in the ED-run Observation Medicine Unit.

## Discussion

The transition from traditional noninvasive cardiac evaluation methods to CCTA in an ED-run Observation Medicine Unit has been examined in several studies, each corroborating the findings of our study regarding reduced length of stay and improved diagnostic accuracy. Multicenter randomized trials demonstrate that CCTA significantly reduces the ED length of stay and hospital length of stay compared to traditional care, without compromising safety or diagnostic outcomes [8,12]. 

Moreover, the high negative predictive value (NPV) and positive predictive value (PPV) of CCTA are well-documented in the literature. The high diagnostic accuracy of CCTA allows for the rapid exclusion of significant CAD and reduces the need for more invasive testing [13]. The ability of CCTA to provide detailed anatomical information about coronary arteries is a significant advantage over traditional stress tests, which often require multiple steps and prolonged observation. When comparing CCTA and FFR-CT to other types of stress tests, several factors need to be considered, including radiation exposure, diagnostic capabilities, and the type of information provided. CCTA typically has a radiation dose ranging from 1 to 12 mSv, while FFR-CT, which uses the same data from CCTA, does not add significant additional radiation exposure. In contrast, exercise stress tests and stress echocardiography involve no radiation, making them suitable for patients where radiation minimization is critical. However, these tests primarily provide functional information on how well the heart handles physical stress, without detailed anatomical visualization. Nuclear stress tests, such as SPECT and PET, involve higher radiation doses, approximately 9-12 mSv for SPECT and 3-7 mSv for PET. These tests provide both functional and perfusion data but at the cost of higher radiation exposure. In comparison, CCTA and FFR-CT offer comprehensive anatomical and functional insights with potentially lower or comparable radiation doses, especially with modern low-dose protocols. Thus, CCTA and FFR-CT stand out for their detailed coronary imaging capabilities, balancing detailed anatomical and functional information with relatively efficient radiation management.

The provider and nursing adaptation process observed in our study is consistent with findings from other institutions. Educational programs targeting healthcare providers and nurses are crucial for the successful implementation of new clinical protocols. Continuous medical training helps to overcome initial resistance to new diagnostic techniques and ensure adherence to updated clinical guidelines [14].

In terms of clinical outcomes, our findings that no patients undergoing CCTA experienced MACEs are supported by previous research. Patients assessed with CCTA had similar or better outcomes compared to those undergoing traditional evaluation methods, with a low incidence of MACEs at follow-up [15]. The reduction in recidivism rates and the identification of significant findings requiring intervention also align with the benefits of CCTA reported in the literature.

Overall, our study contributes to the growing body of evidence supporting the integration of CCTA into ED protocols for the evaluation of patients with suspected ACS. It highlights the challenges of introducing this pathway into an already-existing ED-run Observation Medicine Unit and methods to overcome those challenges. Future research should focus on prospective studies with larger and more diverse patient populations to further validate these findings and optimize patient selection criteria for CCTA.

Limitations 

This study has several limitations that must be acknowledged. The retrospective design inherently carries risks of selection bias and incomplete data capture. Additionally, the exclusion criteria for CCTA, such as age and preexisting conditions, limit the generalizability of the findings to broader patient populations. Future prospective studies with larger and more diverse cohorts are needed to validate these results and refine patient selection criteria.

## Conclusions

The transition to CCTA for routine noninvasive cardiac evaluation in the ED-run Observation Medicine Unit has demonstrated significant benefits in terms of efficiency and diagnostic accuracy. The implementation of CCTA has led to effective patient stratification with minimal MACEs within 28 days. This study further supports the efficacy and safety of CCTA as the return visits to the ED were primarily for noncardiac issues or scheduled follow-ups, underscoring the reliability of CCTA in initial patient assessment. Further, the reduction in length of stay and potential improvements in clinical outcomes seen in this study highlight the value of CCTA as a superior alternative to traditional stress-testing methods. While proven to be beneficial, the implementation of CCTA in the ED setting may encounter barriers. This study demonstrates that through re-education of both providers and nursing staff, adherence to and use of CCTAs can be sustained and improved, ultimately leading to reduced length of stay. Future research should focus on long-term outcomes and further optimization of patient selection criteria to maximize the benefits of this advanced diagnostic tool.
